# A systematic review and meta-analysis of the efficacy of addition and reduction of Shixiao powder in the treatment of coronary heart disease

**DOI:** 10.3389/fcvm.2026.1699481

**Published:** 2026-02-27

**Authors:** Siyuan Chen, Hui Shen, Yingying Su, Cihang Zhou, Yaping Li

**Affiliations:** 1 Tongde Hospital of Zhejiang Province Affiliated to Zhejiang Chinese Medical University (College of Integrated Traditional Chinese and Western Medicine Clinical Medicine), Zhejiang Chinese Medical University, Hangzhou, Zhejiang, China; 2 Department of Outpatient Office, Tongde Hospital Affiliated to Zhejiang Chinese Medical University (Tongde Hospital of Zhejiang Province), Zhejiang, China

**Keywords:** angina dectoris, coronary heart disease, curative effect, meta-analysis, Shixiao powder

## Abstract

**Objective:**

To evaluate the effectiveness and safety of combining Shixiao Powder with Western medicine for treating coronary heart disease-induced angina.

**Methods:**

The databases CNKI, VIP, Wanfang, SinoMed, PubMed, Embase, Web of Science, and Cochrane were searched until March 19th, 2024 for randomized controlled studies on the treatment of angina pectoris in coronary heart disease using Shixiao Powder combined with Western medicine. Quality assessment was conducted using the Cochrane Collaboration's tool. Heterogeneity was tested using the Chi-squared-based Q statistic test and I2 statistic.

**Results:**

The study included a total of 15 articles, with 1204 patients in the cohort, divided into 625 cases in the observation group and 579 cases in the control group. Compared to Western medicine alone, the combination of Shixiao powder and Western medicine significantly improved the effective rate in treating angina pectoris of coronary heart disease [RR = 1.25, 95%Cl (1.18–1.32), *P* < 0.00001]. The improvement in ECG ST segment was superior [RR = 1.32, 95%Cl (1.21–1.43), *P* < 0.00001]. Additionally, there was a decrease in serum CRP level [WMD=−0.8, 95%Cl (-1.43–0.18), Z = 2.52, *P* = 0.012 < 0.05]. The assessment of publication bias concluded that there was no publication bias, suggesting that the conclusions of this study are accurate and reliable.

**Conclusion:**

The current evidence supports that combining Shixiao Powder with Western medicine effectively and safely treats angina pectoris associated with coronary heart disease.

**Systematic Review Registration:**

https://www.crd.york.ac.uk/PROSPERO/recorddashboard, PROSPERO CRD42024537794.

## 1 Introduction

Coronary heart disease (CHD) is a heart disease caused by the narrowing or occlusion of the lumen due to coronary atherosclerosis, which leads to myocardial ischemia, hypoxia or necrosis. When lumen stenosis >50%, angina pectoris caused by transient myocardial insufficient blood supply and oxygen supply is easy to occur (1). CHD occurs more often in middle-aged and elderly people, and more males than females (2). According to the Global Burden of Disease International Collaborative Study 2023, the global prevalence of coronary heart disease is 3,610.2 per 100,000, and the number of people with the disease worldwide is estimated at 320 million. The estimated global mortality count attributed to coronary heart disease stands at 9.239 million, with an age-standardized mortality rate of 108.8 per 100,000 individuals. This condition remains the leading cause of death worldwide (3).In the latest guidelines for the treatment of coronary heart disease, western medicine mainly includes lifestyle management, pharmacological treatment, and surgical treatment, which focuses on improving ischemia, relieving chest pain, preventing myocardial infarction, and improving prognosis (4). Lifestyle management mainly includes methods such as Tobacco abstinence, a balanced diet, and regular exercise. About pharmacological treatment, it mainly includes antithrombotic therapy, lipid-lowering therapy and beta-blockers therapy. Among these pharmacological treatment, antithrombotic therapy is the cornerstone in the management of coronary heart disease. It is often recommended 12-months dual antiplatelet therapy(DAPT), which is consist of a potent P2Y12 receptor inhibitor in addition to aspirin. Lipid-lowering therapy can improve the prognosis of coronary heart disease, which goal is to lower LDL-C to <1.4 mmol/L and to achieve a ≥ 50% LDL-C reduction from baseline. Surgical treatments mainly include Percutaneous Coronary Intervention(PCI) and Coronary Artery Bypass Grafting(CABG). However, due to the complex condition and long course of the disease, long-term medication may cause adverse reactions (1). Many patients are troubled by the side effects of drugs, such as muscle pain and weakness (5), liver and kidney function damage (6), and bleeding (7). Moreover, a considerable number of patients will develop drug resistance, which is frequently observed in antithrombotic therapy. Additionally, postoperative patients may experience anxiety, depression and chest tightness.

Shixiao Powder is derived from *Bureau of Peaceful Benevolent Dispensary* of the Song Dynasty in China. It is composed of the equal amount of Typhae Pollen and Trogopterori Faeces. It has the effect of activating blood and resolving stasis, subduing swelling to relieve pain (8). Modern pharmacology has proved that Typhae Pollen has the effects of anti-inflammation, promoting angiogenesis, hemostasis and anti-thrombosis (9–11), while Trogopterori Faeces has the effect of reducing inflammatory reaction (11–13). It has been found that Shixiao Powder combined with conventional Western medicine has a certain effect on angina pectoris of coronary heart disease, but the existing evidence on its combination with western medicine is characterized by inconsistent quality, contradictory findings, or a lack of high-level synthesis. This study aims to determine whether adding Shixiao Powder to conventional Western medicine is more effective in improving symptoms and objective indicators in patients with angina pectoris due to CHD, without increasing adverse events, by synthesizing evidence from RCTs. This study uses Meta-analysis to systematically evaluate the efficacy and safety of Shixiao Powder combined with conventional Western medicine in the treatment of angina pectoris of coronary heart disease, to provide evidence-based medical evidence for clinical application and treatment plan formulation.

## 2 Materials and methods

### 2.1 Data source and retrieval strategy

Chinese databases (CNKI, VIP, CBM, WanFang) and English databases (PubMed, Embase, Web of Science and Cochrane), WHO trial registry as well as ClinicalTrials.gov, to facilitate finding ongoing or unpublished literature. The search time was from the establishment of each database to March 19, 2024. Chinese search terms included “Guan Xin Bing” “Xiong Bi” “Xin Jiao Tong” “Shixiao San”, etc. English search terms included “Coronary Disease” “Diseases, Coronary Heart” “Angina pectoris” “Stenocardia” “Shixiao Powder” and so on. A combination of subject words and free words was used for retrieval.

### 2.2 Inclusion criteria

#### 2.2.1 Study types the languages of RCT were limited to Chinese or English

The subjects were patients with coronary atherosclerotic heart disease and angina pectoris, regardless of sex, age, and course of disease. The diagnostic criteria were established using *the Guidelines for the diagnosis and treatment of stable coronary artery disease* (14) and *the 2019 ESC Guidelines for the diagnosis and treatment of stable coronary artery disease management of chronic coronary syndromes (*4). Clinical diagnostic criteria: (1) patients with coronary artery atherosclerosis and stenosis confirmed by coronary angiography; (2) Post-sternal compression pain or suffocation occurred. The pain lasted from a few minutes to more than 10 min, and nitrate drugs could relieve the pain. (3) Chest discomfort can radiate from the precordial area to the left shoulder, left arm, ring finger and little finger, or to the neck, pharynx or mandible. (4) It is often induced by fatigue, emotional excitement and cold (14).

#### 2.2.2 Interventions

The control group followed the *guidelines for the Rational Use of Drugs for Coronary Heart Disease* (1) and used conventional Western medicines such as acid esters, *β*-blockers, calcium channel blockers, aspirin, clopidogrel, statins, angiotensin-converting enzyme inhibitors, etc. For patients with basic chronic diseases such as hypertension and diabetes, antihypertensive drugs and hypoglycemic drugs should be added to reduce the influence of non-coronary heart disease on the outcome indicators. The intervention group was treated with Shixiao Powder based on the control group. Both groups did not limit the dose and course of treatment.

#### 2.2.3 Outcome measures

The primary outcome measure was the effective rate of angina reduction. The secondary outcome indicators included the improvement of the electrocardiogram ST segment, adverse reactions, and TCM syndrome scores. “Effective” angina reduction was defined as the absence of a 50% or greater reduction in the number of angina episodes or a one-grade improvement in the degree of angina without causing the same level of exertion (15, 16). “Effective” ST-segment improvement was defined as the recovery of the ischemic ST-segment in resting electrocardiogram, or the ischemic ST segment of resting electrocardiogram rose more than 1.5 mm before treatment but did not return to the normal level, or the inverted T wave in main lead became more than 50% shallow or the T wave changed from flat to upright (15). “Effective” of the TCM symptom score was defined as a ≥ 30% decrease in the patient's TCM symptom score. Effective rate=number of effective cases/sample size  × 100%.

### 2.3 Exclusion criteria

Duplicate publications; Literature with full text or valid data was not available.

### 2.4 Literature screening

The retrieved literature was imported into Endnote, and the initial screening was completed according to the bibliographic information after removing duplicates. The primary screening literature was then read in full to determine whether it was finally included. The screening task was carried out by two researchers individually, and any discrepancies that arose were resolved through collaborative deliberation. In case of disagreement, the third researcher made the decision.

### 2.5 Data extraction

A data extraction table was redesigned, including the name of the first author, publication time, sample size, age, gender, intervention measures, treatment course, and outcome indicators. Two researchers were required to complete the extraction work independently, and the third researcher was invited to decide in case of disagreement.

### 2.6 Quality evaluation

The Cochrane Risk of Bias tool was utilized by two independent reviewers to evaluate the potential bias in the included studies, and a thorough examination of the studies was performed for verification purposes. If there was any disagreement, the third reviewer was consulted. The risk of bias was assessed in seven domains: random sequence generation, allocation concealment, blinding of participants and personnel, blinding of outcome assessors, incomplete outcome data reporting, selective outcome reporting, and other sources of bias.

### 2.7 Statistical analysis

The meta-analysis was conducted using Stata 17 and RevMan 5.4.1 software. Relative risk (RR) was employed as the effect measure for binary variables, while weighted mean difference (WMD) was utilized when continuous variables shared the same unit of measurement. The confidence interval (CI) used in this analysis had a level of significance set at 95%. The size of statistical heterogeneity was judged by I^2^ and *P* value, I^2^ > 50% and *P* < 0.1 of the Q test indicated large heterogeneity, and I^2^ ≤ 50% and *P* > 0.1 of the Q test indicated small heterogeneity. Publication bias was determined by funnel plot and Egger test. If *P* < 0.05 indicated publication bias, stata15.1 software was used for sensitivity analysis and subtraction method to evaluate the stability of the results.

## 3 Results

### 3.1 Literature search

**Figure 1:**
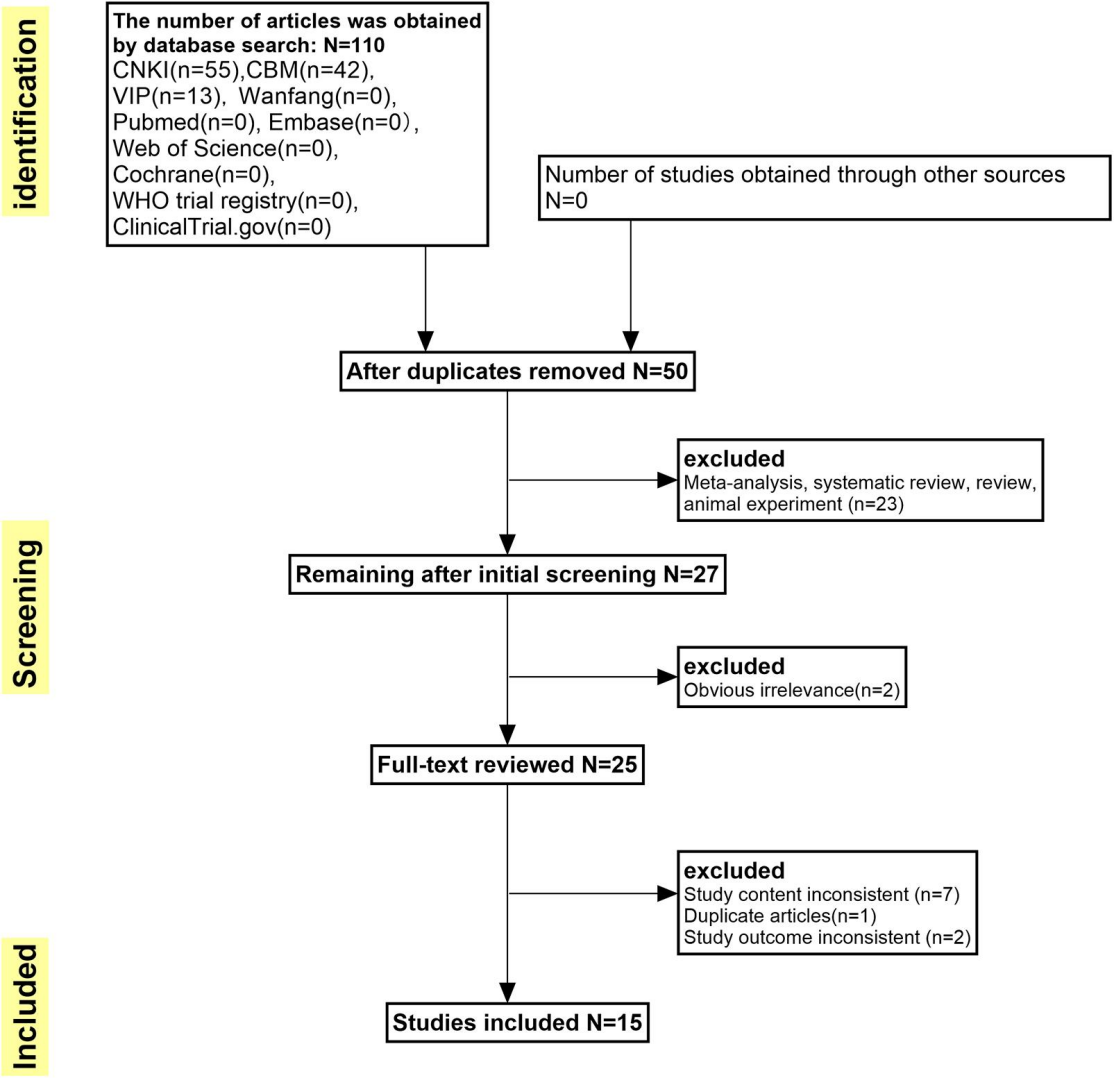
Flow chart of literature search.

After the application of inclusion and exclusion criteria, a total of 110 articles written in Chinese language were acquired. A total of 60 articles with duplicate content were eliminated, while 23 animal experiments were excluded based on the evaluation of their titles and abstracts. Additionally, 2 studies were removed due to inconsistent research content, and a further 10 studies were excluded after thorough examination of the full text. Ultimately, a total of 15 studies met the inclusion criteria Figure 1.

The study encompassed fifteen randomized controlled trials (9, 10, 16–28), involving a total of 1,204 patients, with 625 assigned to the intervention group and 579 to the control group. The sample size of the included literature ranged from 21 to 64 patients, and the treatment duration ranged from 10 to 56 days. The intervention group in all the studies included received conventional Western medicine for coronary heart disease, with the incorporation and exclusion of Shixiao Powder as an interventional approach. Three articles (10, 16, 17) analyzed that the TCM syndrome of the included population was Qi deficiency-induced blood stasis pattern. Five (10, 17–19, 25) used TCM syndrome score as one of the outcome indicators. The included literature pointed out that the baseline conditions of the patients such as age, gender, and disease progression were comparable. The fundamental characteristics of the literature included are presented in Table 1.

**Table 1:** Fundamental characteristics.

Article ID	Sample Size		male/Female		Age range (average)		Treatment Methods		Treatment (d)
	IG	CG	IG	CG	IG	CG	IG	CG	
Chengxue Yu	50	50	30/20	28/22	62.8 ± 6.0	65.6 ± 7.3	SXP + CWM	CWM	14
Dan Peng	50	50	31/19	29/21	64.2 ± 5.8	66.3 ± 7.1	SXP + CWM	CWM	14
Fei Ying	36	35	15/21	16/19	80.19 ± 4.59	79.94 ± 4.16	SXP + CWM	CWM	30
Hui Chen	60	56	32/28	31/25	58.3 ± 6.8	57.9 ± 6.3	SXP + CWM	CWM	10
Huipeng Zhang	25	25	14/11	13/12	55 ± 3.6	53 ± 2.6	SXP + CWM	CWM	28
Jingjun Li	68	55	35/33	30/25	57.0 ± 5.6	56.0 ± 4.8	SXP + CWM	CWM	21
Kaiqiang Duan	64	64	34/30	33/31	58.92 ± 8.84	57.28 ± 9.72	SXP + CWM	CWM	25
Liwu Qu	36	30	25/11	21/9	54∼81	57∼79	SXP + CWM	CWM	28
Weiming Zhang	33	30	23/10	21/9	67.4 ± 6.91	64.89 ± 7.98	SXP + CWM	CWM	28
Xiantian Gong	35	35	23/12	24/11	57.85 ± 8.94	58.27 ± 8.25	SXP + CWM	CWM	28
Yan Xu	30	30	10/20	17/13	66.40 ± 10.52	64.53 ± 13.54	SXP + CWM	CWM	56
Ying Chen	30	30	12/18	14/16	40∼69	40∼69	SXP + CWM	CWM	28
Yongzhi Zhao	36	38	18/18	21/17	49∼66	46∼65	SXP + CWM	CWM	30
Yufeng Liu	30	30	15/15	14/16	41∼72	40∼73	SXP + CWM	CWM	30
Zhongjian Wang	42	21	36/27		45∼68		SXP + CWM	CWM	30–45

IG, intervention group; CG, control group; SXP, Shixiao Powder; CWM, conventional Western medicine.

### 3.2 Literature quality assessment

**Figure 2:**
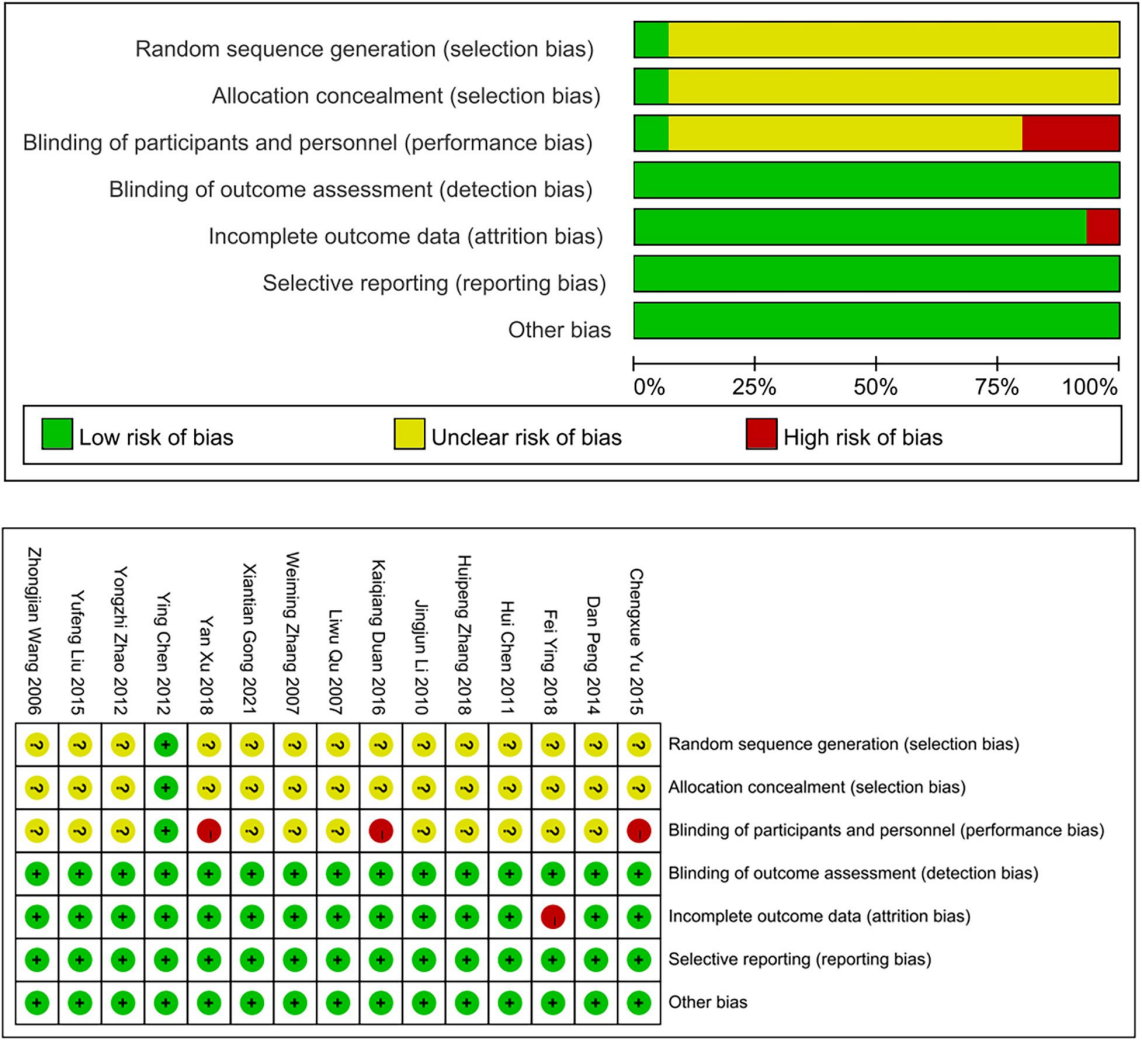
Quality assessment judged by Cochrane Collaboration's tool.

The fifteen included literature (9, 10, 16–28) were all randomized controlled studies, demonstrating a high level of methodological rigor. Notably, one study (18) employed the random number table method and was deemed to have a low risk of bias. Fourteen (9, 10, 16, 17, 19–28) did not specify the randomization method, and the risk of bias was not clear. One article (18) used single-blind and was rated as low risk of bias. Three articles (10, 25, 26) proposed to sign informed consent with patients, judged as unblinded and rated as high risk. The remaining studies did not explicitly state whether they were blinded or not, and the risk of bias was rated as unclear. Seven cases were lost to follow-up in one article (17), which was judged as incomplete result data and rated as a high risk of bias. The remaining included articles did not drop out and were rated as low risk of bias. The risk of bias was assessed in Figure 2.

### 3.3 Results of meta-analysis

#### 3.3.1 Effective rate of angina pectoris reduction

Fifteen research studies (9, 10, 16–28) documented the rate of successful reduction in angina symptoms, encompassing a combined sample size of 1,204 individuals. This included 625 participants in the experimental group and 579 participants in the control group.

**Figure 3:**
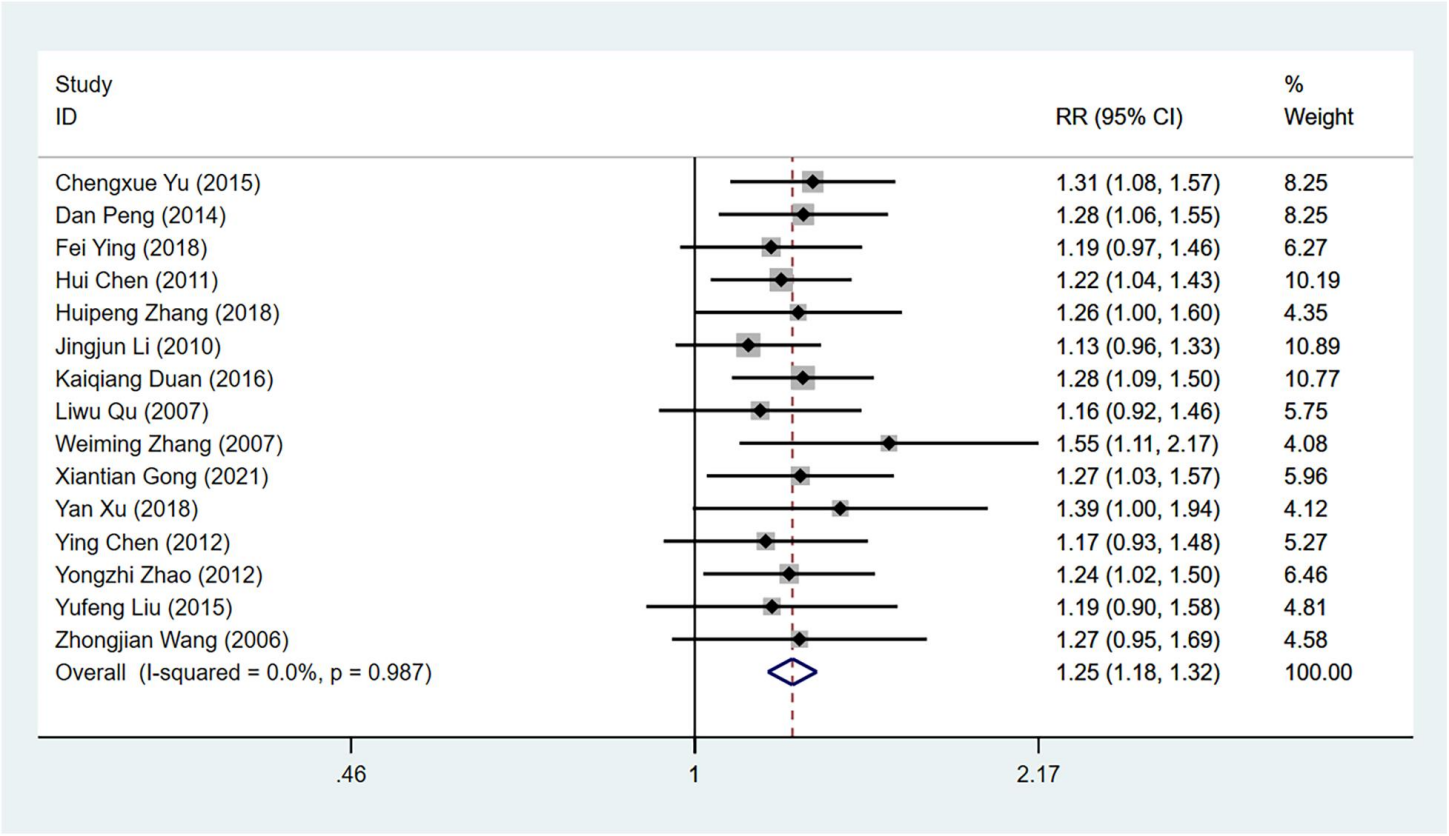
Forest plot illustrating the efficacy of Shixiao Powder combined with western medicine versus western medicine alone in alleviating angina symptoms.

The test results indicated that the studies did not exhibit any statistically significant heterogeneity (I^2^ = 0% < 50%, *P* = 0.99 > 0.1 by Q test), leading to the adoption of the fixed effect model for conducting Meta-analysis. The findings indicated that the concurrent utilization of Shixiao Powder alongside Western medicine demonstrated enhanced effectiveness in managing angina pectoris, surpassing the efficacy of Western medicine alone by 1.25 times [RR = 1.25, 95%Cl (1.18–1.32), Z = 7.84, *P* < 0.00001 < 0.05]. This disparity exhibited statistical significance Figure 3.

**Figure 4:**
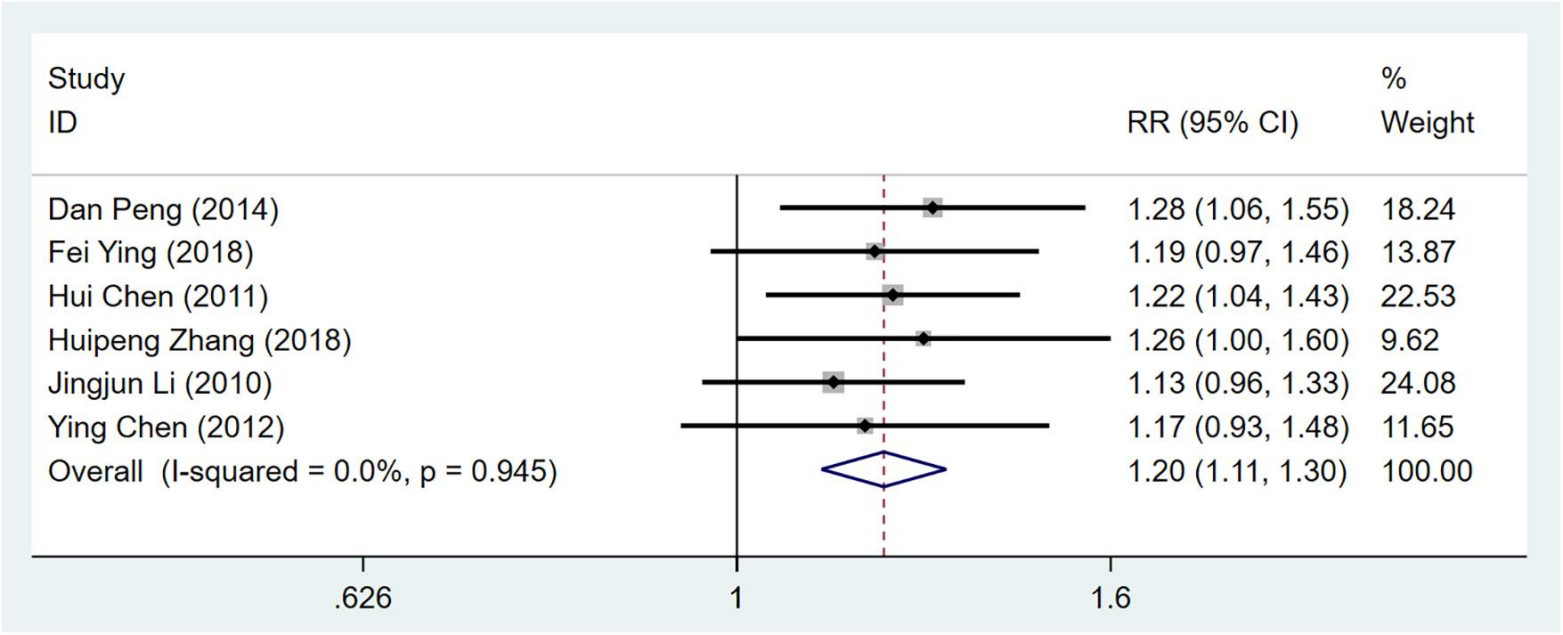
Subgroup analysis forest plot illustrating the efficacy of selected similar western medicine study treatments in alleviating angina symptoms.

Due to the imbalance caused by the different Western medicines used in the control groups across various studies, a supplementary subgroup analysis was conducted using a combination of four types of Western medicines in the control group: antiplatelet agents, statins, *β*-blockers, and nitrates. Six studies (9, 16, 18, 20, 22, 29) involving 520 patients were included, with 269 in the observation group and 251 in the control group. The test results indicated no statistically significant heterogeneity among the studies (I^2^ = 0%<50%, *P* for the Q test=0.95 > 0.1), so a fixed-effects model was chosen for the meta-analysis. The results indicate that when the use of Western medicine is restricted to these four categories, the efficacy in the observation group remains superior to that of the control group (1.20 times), with a statistically significant difference [RR = 1.20, 95% CI (1.11–1.3), Z = 4.61, *P* < 0.00001 < 0.05], consistent with the previous analysis Figure 4.

#### 3.3.2 ECG ST segment improvement

**Figure 5:**
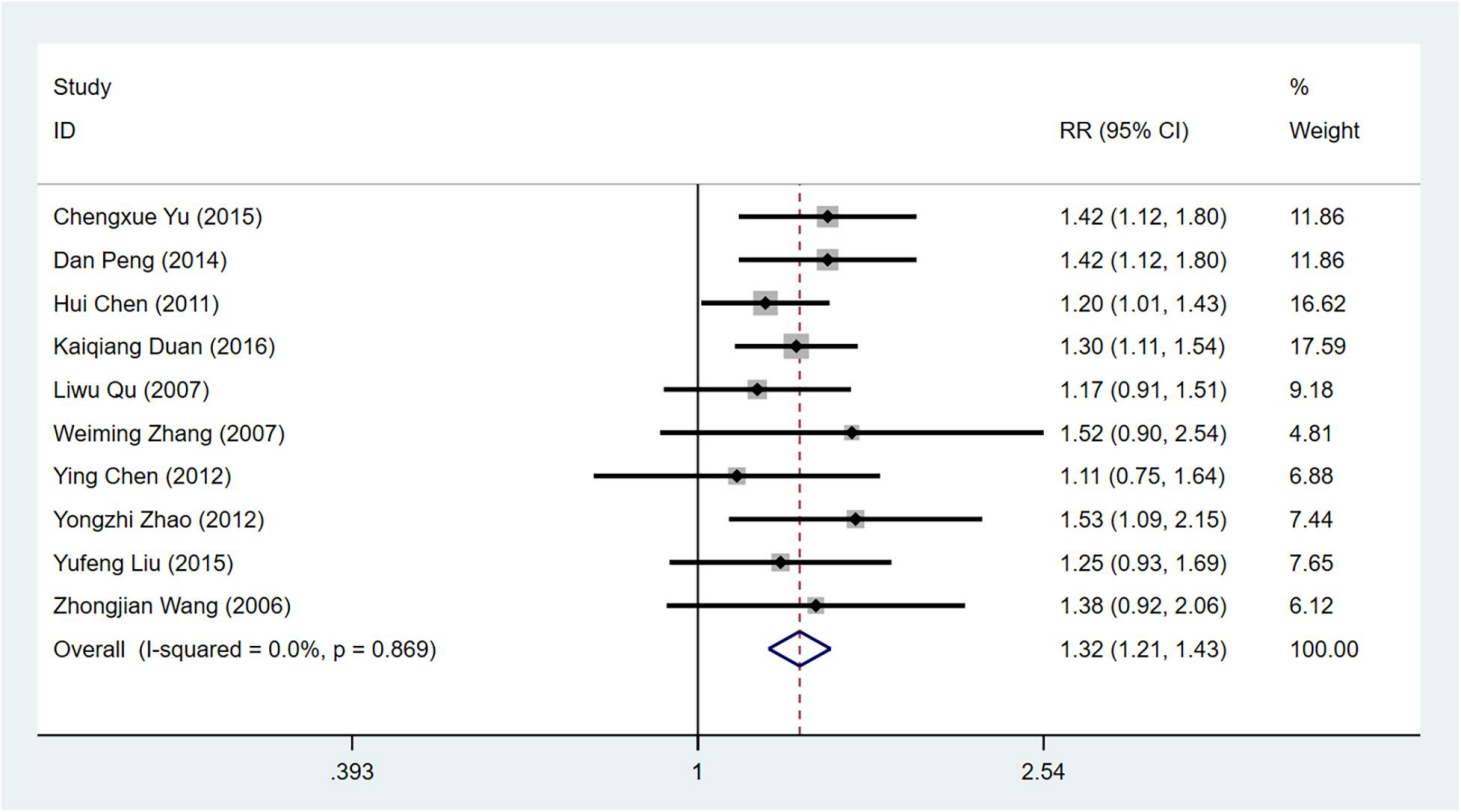
Forest plot illustrating the efficacy of Shixiao Powder combined with western medicine versus western medicine alone in enhancing the ECG ST segment.

The enhancement of the ECG ST segment was documented in ten research papers (10, 16, 18, 21–24, 26–28), encompassing a combined sample size of 830 individuals. Among these participants, 431 were assigned to the intervention group while 399 belonged to the control group. The heterogeneity test results revealed that the observed variability among the literature chosen for this study did not achieve statistical significance [I^2^ = 0% < 50%, *P* = 0.869 > 0.1 of Q test], thus prompting the utilization of a fixed effect model for conducting Meta-analysis. The findings indicated that the utilization of Shaoxiao Powder in conjunction with Western medicine demonstrated enhanced effectiveness in enhancing the ECG ST segment compared to solely relying on Western medicine. The combined treatment exhibited a curative effect 1.32 times greater than using Western medicine alone [RR = 1.32, 95%Cl (1.21–1.43), Z = 6.43, *P* < 0.00001 < 0.05], and this disparity was statistically significant Figure 5.

#### 3.3.3 Effective rate of TCM symptom score

Five studies (10, 17–19, 25) examined the efficacy of TCM symptom score in a sample size of 389 patients, with 195 individuals in the intervention group and 194 individuals in the control group.

**Figure 6:**
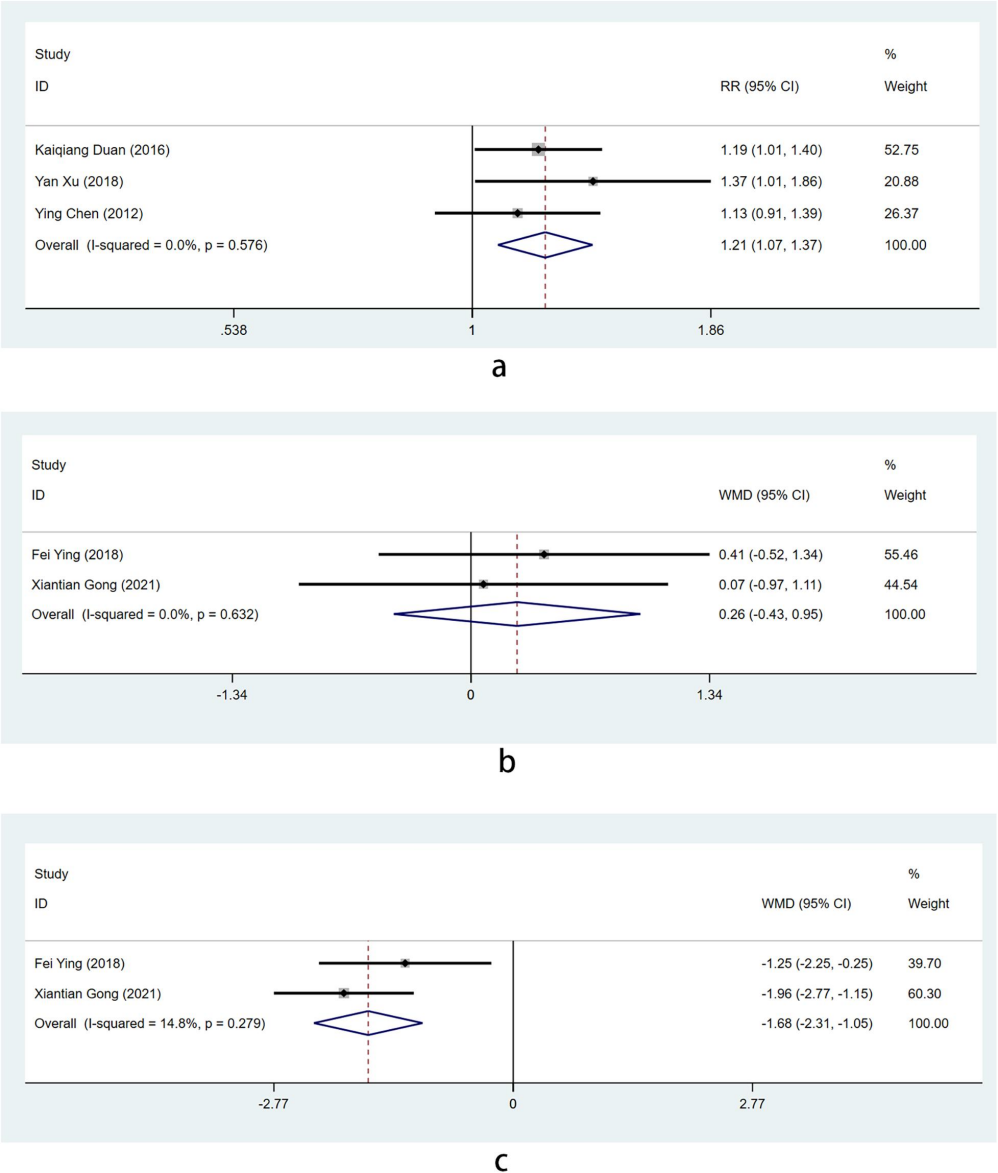
Forest plot illustrating the efficacy of Shixiao Powder combined with western medicine versus western medicine alone in effective rate of TCM symptom score.

Among them, three articles (10, 18, 25) utilized dichotomous variables to report on a total of 248 patients (intervention group: *n* = 124; control group: *n* = 124). The heterogeneity test indicated that the selected literature in this study did not exhibit statistically significant heterogeneity [I^2^ = 0% < 50%, *P* = 0.576 > 0.1 for Q test], therefore, the fixed-effect model was used for meta-analysis. The findings revealed that combining Shixiao Powder with Western medicine resulted in significantly better efficacy compared to Western medicine alone, showing an effect size 1.209 times greater than the latter and this difference was found to be statistically significant [RR = 1.209, 95%Cl (1.07–1.37), Z = 3.02, *P* = 0.002 < 0.05] Figure 6A.

Two articles (17, 19) were reported using continuous variables, involving a total of 141 patients, including 71 in the intervention group and 70 in the control group. Firstly, There was no heterogeneity in the baseline period effect size between the two groups (I^2^ = 0% < 50% and *P* = 0.632 > 0.1 by Q test). The fixed effect combined with the baseline effect size was selected, and the results showed that there was no difference in the TCM symptom score between the two groups at baseline (WMD=0.26; Z = 0.73, *P* = 0.463 > 0.05) Figure 6B.

After conducting a test for heterogeneity, it was found that there was no significant variation among the selected literature in this study [I^2^ = 14.8% < 50%, *P* = 0.279 > 0.1 by Q test]. Fixed effects were employed for subsequent meta-analysis of effect size. The findings indicated that the combination of Shixiao Powder and Western medicine exhibited superior efficacy compared to Western medicine alone, with a curative effect 1.68 times higher than the latter, and this difference was statistically significant [RR = −1.68, 95%Cl (-2.31–1.05), Z = 5.23, *P* < 0.0001 < 0.05] Figure 6C.

#### 3.3.4 C-reactive protein levels

**Figure 7:**
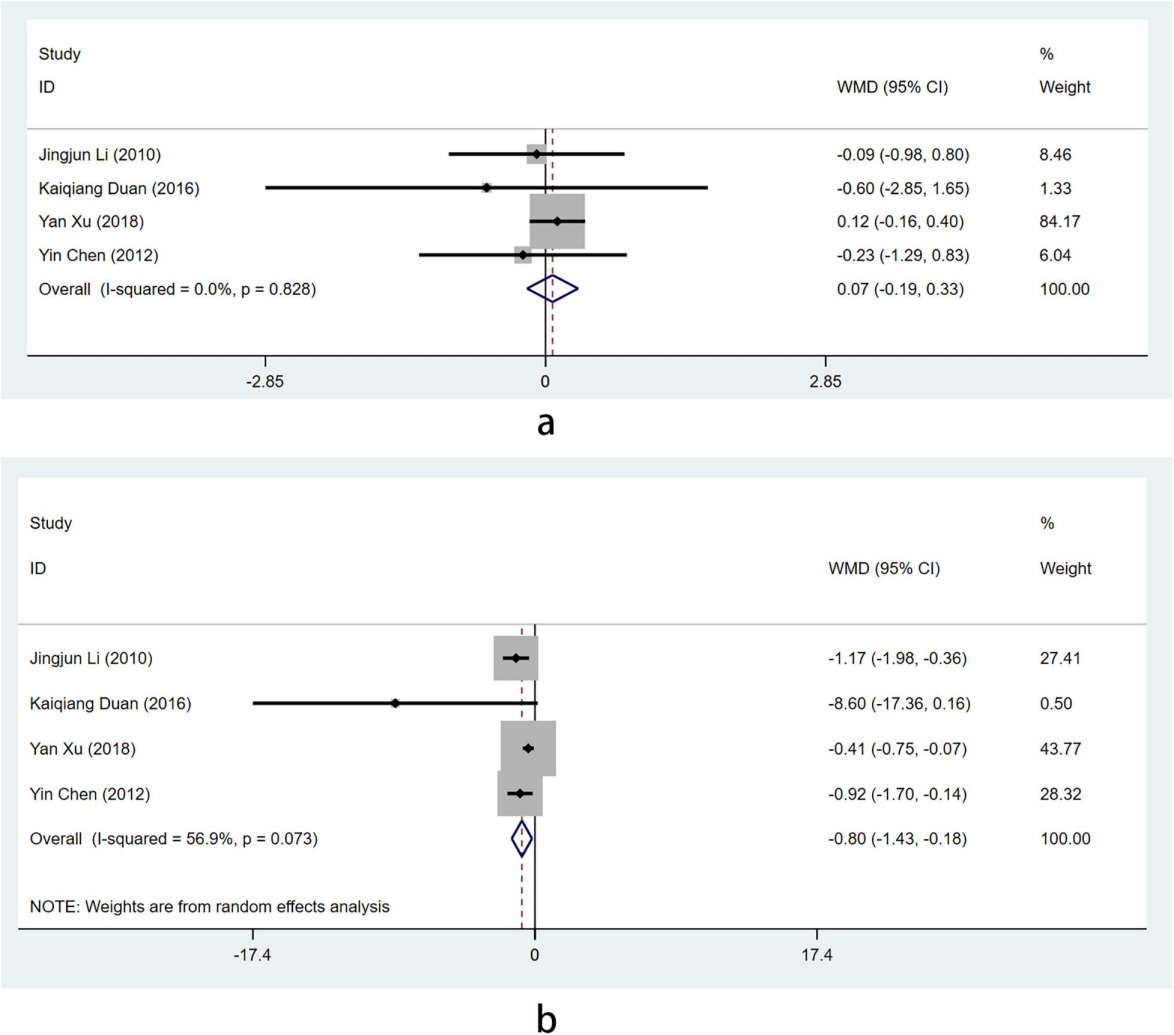
Forest plot illustrating the efficacy of Shixiao Powder combined with western medicine versus western medicine alone in C-reactive protein levels.

Four studies (10, 18, 20, 25) were conducted on C-reactive protein (CRP), with a total of 371 patients participating. Among them, the intervention group consisted of 192 cases while the control group had 179 cases. There was no observed variation in effect size between the two groups when utilizing the baseline period test (I^2^ = 0% < 50% and *P* = 0.828 > 0.1 by Q test). By selecting the fixed effect combined with the baseline effect size, it can be concluded that there is no discernible disparity in CRP levels between the two groups at baseline (WMD=0.071, Z = 0.54, *P* = 0.590 > 0.05) Figure 7A. A subsequent meta-analysis could potentially be conducted.

After conducting a heterogeneity test, it was indicated that there existed diversity among the types of literature chosen for this study [I_2_ = 56.9% > 50%, and *P* = 0.073 < 0.1 based on Q test]. Therefore, the random effect model was employed for Meta-analysis. The results showed that CRP in the intervention group after treatment was significantly lower than that in the control group [WMD=−0.8, 95%Cl (−1.43–0.18), Z = 2.52, *P* = 0.012 < 0.05] Figure 7B.

#### 3.3.5 Adverse reactions

Four studies (10, 17, 24, 27) reported adverse reactions, of which two studies (17, 24) reported specific adverse reactions, but the symptoms were mild or relieved spontaneously with medication. The other two studies reported no adverse reactions during the treatment Table 2**.** It is suggested that Shixiao Powder is safe and has no obvious adverse reactions when combined with Western medicine.

**Table 2:** Adverse reactions.

Article ID	Sample Size		Adverse Reaction (case)	
	IG	CG	IG	CG
Kaiqiang Duan	64	64	0	0
Zhongjian Wang	42	21	2 dizziness	3 Headache; 3 Dizziness; 5 Facial flushing
Fei Ying	36	35	1 ELE; 1 mild cough; 1 diarrhea	2 ELE; 1 mild cough
Weiming Zhang	33	30	0	0

IG, intervention group; CG, control group; ELE, Edema of the lower extremities.

#### 3.3.6 Risk of bias

**Figure 8:**
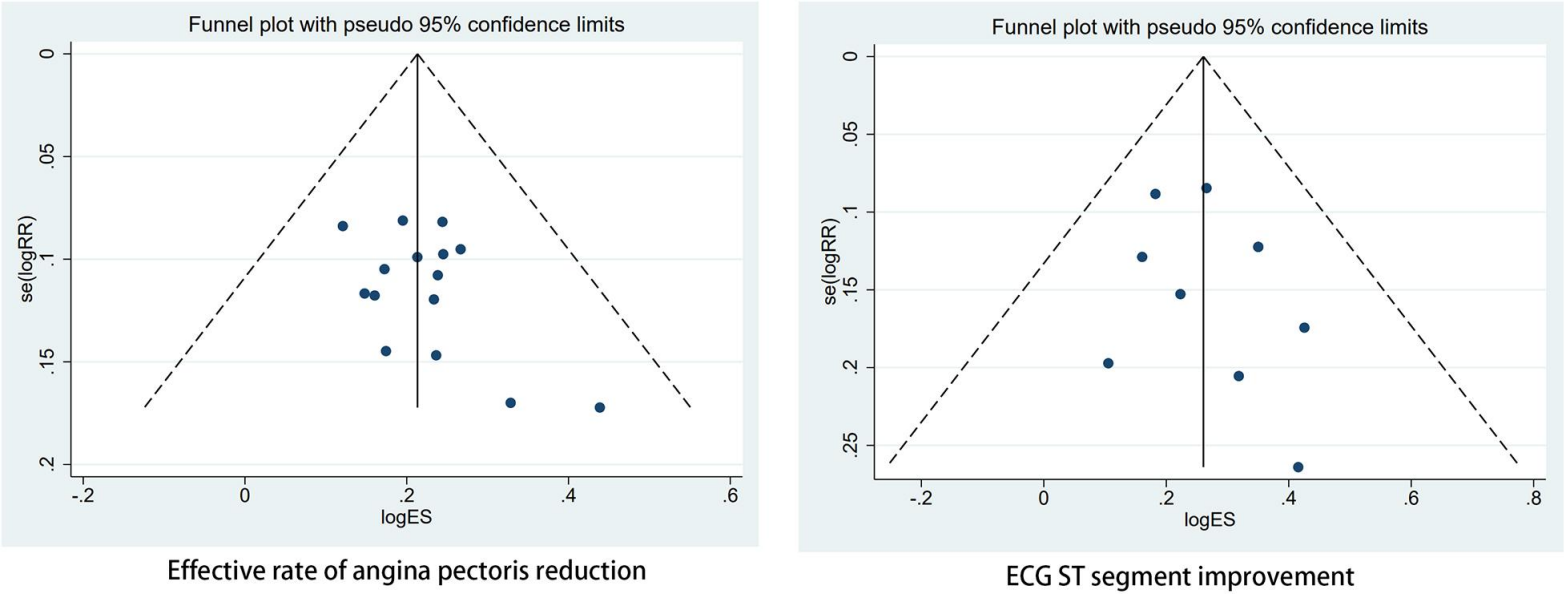
Funnel plots of effective rate of angina pectoris reduction and ECG ST segment improvement.

**Figure 9:**
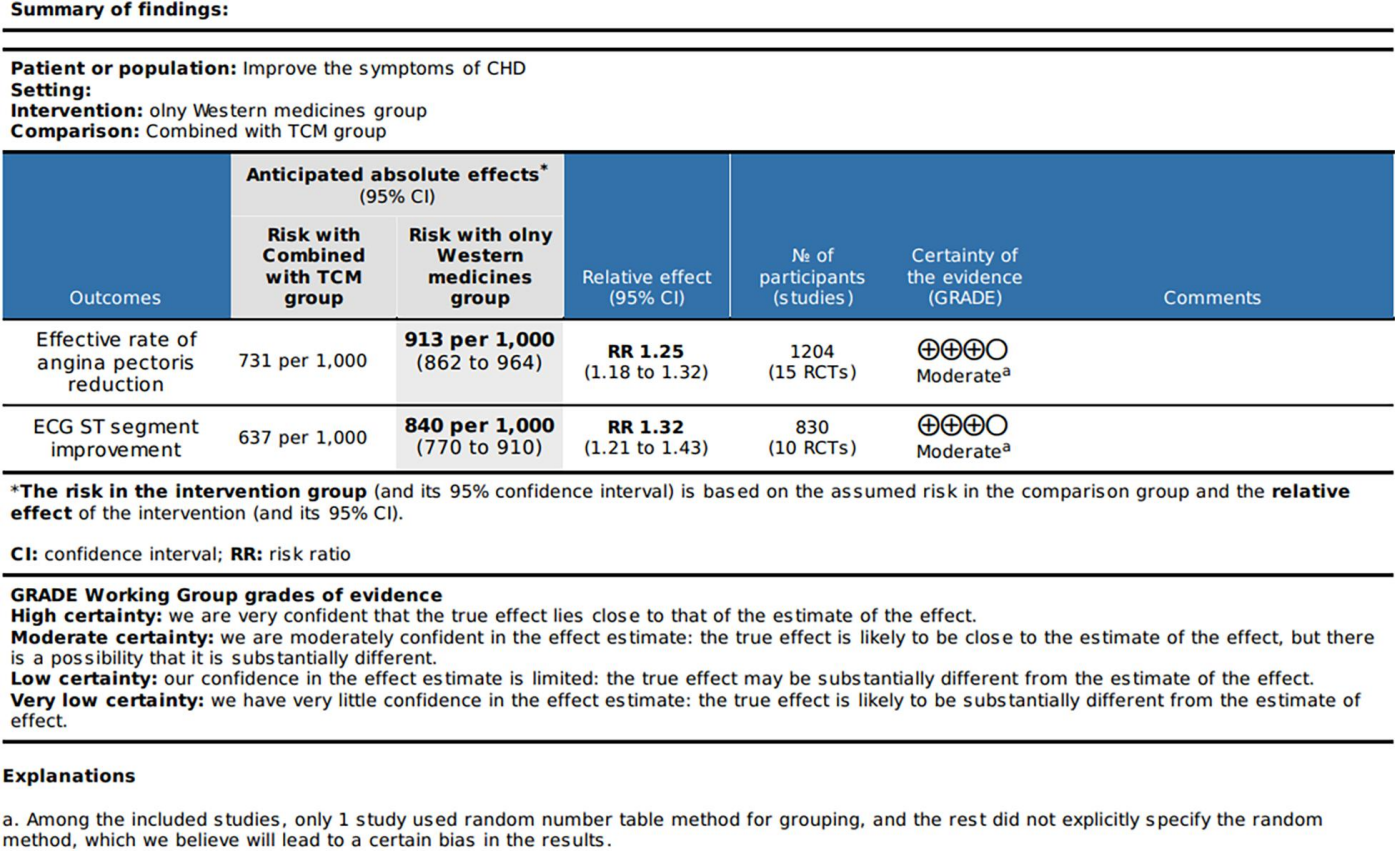
Quality grade.

The effective rate of angina pectoris reduction and ECG ST segment improvement underwent publication bias analysis, revealing a symmetrical scatter distribution in the funnel plot (Egger's test indicated *P* = 0.109 > 0.05, Egger's test *P* = 0.413 > 0.05) Figure 8. These findings suggest the absence of publication bias and validate the accuracy and reliability of this study's conclusion.The quality grade indicates that after accounting for the overall risk of bias, imprecision, inconsistency, indirectness, and publication bias, the reliability of the evidence for the aforementioned two items is “moderate” Figure 9.

## 4 Discussion

Epidemiological studies have shown that the prevalence and mortality of coronary heart disease is on the rise year by year (3). As a major public health concern, CHD increases the social economic burden and threatens human health. Conventional western medicine mostly relies on various kinds of drugs to prevent disease progression, but there are some potential risk factors such as aspirin resistance, gastrointestinal adverse effects and the risk of bleeding events. Therefore, it is critical to explore the combination of traditional Chinese and western medicine in treating angina pectoris of CHD.

Angina pectoris of CHD is classified as “Chest bi-impediment” (30, 31) in TCM. In the Ming Dynasty, there were already medical case records of Shixiao Powder to treat chest bi-impediment. Shixiao Powder is composed of Typhae Pollen and Trogopterori Faeces, which have the effect of activating blood and resolving stasis, subduing swelling to relieve pain. Modern pharmacology has proved that Typhae Pollen has the effects of anti-platelet aggregation (32), reducing inflammatory response, regulating blood lipids (13), and analgesia (33). Arabinogalactose extracted from Typhae Pollen can increase the secretion of immunomodulatory factors by activating macrophages (34). The carbonized products of Typhae Pollen can reduce the inflammatory response and oxidative stress in rats with acute kidney injury (35). Interestingly, the carbonized products of Typhae Pollen can significantly reduce activated thromboplastin time and increase blood fibrinogen content and platelet levels in rats (36). Trogopterori Faeces also has the effect of anti-platelet aggregation (32) and reducing inflammation (11). Some clinical studies have found that, compared with the western medicine group, Shixiao Powder with other traditional Chinese medicine formula, such as Xuefuzhuyu Decoction and Gualou Xiebai Banxia Decoction, can improve the effectiveness of drugs, enhance the cardiac function and relieve relevant symptoms. However, there are a limited number of articles on the treatment of angina pectoris due to coronary heart disease with the combination of Western medicine and the single use of Shixiao Powder.

A total of 15 randomized controlled trials (RCTs) involving 1,204 patients were included in this study. The efficacy and safety of Shixiao Powder in the treatment of angina pectoris caused by coronary heart disease were systematically evaluated. The results demonstrated that, compared to conventional Western medicine treatment, modified Shixiao Powder significantly improved the effective rate of angina pectoris, enhanced ECG ST segment improvement, ameliorated TCM clinical syndrome, and reduced CRP levels. Furthermore, the combination therapy with Shixiao Powder did not result in an increase in adverse effects among patients with CHD.

The limitations of this study were as follows: (1) The choice, dosage and treatment course of conventional Western medicine were different in different intervention measures. To mitigate the impact of these differences, we conducted a subgroup analysis of studies using similar Western medicines as a control group, although differences in dosage still exist. (2) The included primary studies did not distinguish or clearly report angina type (tablevs, unstable). This major source of clinical heterogeneity may significantly affect the precision of our pooled estimates and limit the generalizability of conclusions to specific patient subgroups. (3) The results of the publication bias test indicated the absence of publication bias in the included studies. However, only one study suggested implementing single-blind methodology, while three studies proposed conducting unblinded research. Notably, blinding was not mentioned in all other studies, implying potential presence of additional biases (37). CHD is a type of chronic disease that requires long-term treatment and care. Some of the included studies have insufficient treatment durations, which do not help clarify the long-term efficacy, nor do they determine whether the prolonged combined use of traditional Chinese and Western medicine might cause liver and kidney toxicity (37). The safety still needs to be verified (4). The studies included in this Meta-analysis were conducted relatively early, and due to limitations in technical methods and other factors, the evaluation indicators were relatively limited. This suggests that future clinical trials should select multiple specific indicators to help determine efficacy. Therefore, we believe that future researchers, in addition to the aforementioned indicators, should appropriately and selectively incorporate the following examinations in trials to help assess patients' condition, prognosis, and risk of recurrence.

Laboratory tests: (1) Blood lipids: low-density lipoprotein, high-density lipoprotein, cholesterol, apolipoprotein A, apolipoprotein B. Univariable Mendelian randomization analysis (38) showed that genetically predicted low-density lipoprotein (OR1.66;95% CI: 1.49–1.86;*P* < 0.001), triglyceride(OR 1.34;95% CI: 1.25–1.44;*P* < 0.001), and apolipoprotein B(OR 1.73;95% CI: 1.56–1.91;*P* < 0.001) levels were associated with a higher risk of CHD. (2) Renal function: glomerular filtration rate (eGFR). A Mendelian randomization study (39) found that mild to moderate renal dysfunction is associated with the risk of CHD, and assessing renal function can help determine the severity of CHD. Another cross-sectional study (40) found that as eGFR declines, the frequency of advanced atherosclerotic lesions progressively increases(33.6%, 41.7%, 52.3%, and 52.8% for eGFRs ≥60, 45–59, 30–44, and <30 mL/min/1.73 m^2^;*P* = 0.006). (3) Other indices: Triglyceride-glucose (41) [TyG, calculated as fasting triglyceride level (mg/dL)×fasting blood glucose level (mg/dL)/2]. An elevated TyG is a potential marker for poor prognosis in CHD patients.

Non-invasive imaging examinations (14, 42), such as Positron Emission Tomography (PET), Cardiac Magnetic Resonance Imaging (CMR), Transthoracic Doppler Echocardiography (TTDE), and Cardiac Computed Tomography (CT), can help assess local ischemia.

Other tests: stress ECG. When there are no obvious abnormalities in the ECG, a stress ECG should be added to help diagnose and rule out latent ischemic conditions (43).

In addition to supplementary examinations, we also urge future researchers to enhance the level of evidence by using random number tables and blinding methods, and to extend the treatment observation period to 3–6 months or even longer. This will not only help in better assessing the patient's condition and prognosis, clarifying short-term and long-term efficacy, increasing the standardization of such research, and enhancing the reliability of the trial results, but also provide valuable assistance for the subsequent clinical treatment of CHD patients.

## 5 Conclusion

Current evidence suggests that the combination of Shixiao Powder and conventional Western medicine may have a positive impact on angina pectoris attacks, ECG ST segment abnormalities, TCM clinical syndrome, and recurrent coronary heart disease events (44). Publication bias assessment indicated no publication bias, thus confirming the accuracy and reliability of this study's conclusions. This combined approach appears to be more effective than using Western medicine alone while maintaining a good safety profile. However, it is important to note that the available literature in this study has limitations in terms of quality. Future randomized controlled trials investigating the therapeutic potential of Shixiao Powder in CHD should consider incorporating more objective indicators to evaluate both efficacy and safety.

## Data Availability

The original contributions presented in the study are included in the article/Supplementary Material, further inquiries can be directed to the corresponding author.
